# ARE WE WAREHOUSING PEOPLE WITH SERIOUS MENTAL ILLNESS? EXPLORING US NURSING HOMES IN 2000 AND 2021

**DOI:** 10.1093/geroni/igae098.3792

**Published:** 2024-12-31

**Authors:** Kim Hadson, Marissa Bergh, Jasmine Travers

**Affiliations:** NYU Rory Meyers College of Nursing, New York, New York, United States; New York University, Long Island City, New York, United States; New York University, New York, New York, United States

## Abstract

The 1999 Supreme Court Olmstead decision reaffirmed people with serious mental illness (SMI) have the right to care integrated into community settings. The growth of SMI in nursing homes (NHs) suggests care has instead shifted to NHs. Insufficient attention has been given to the facility characteristics and state environments to understand if individuals with SMI in NHs are receiving high quality, equitable care. We used cross-sectional LTCFocus data from 2000 and 2021 to identify NHs with high volumes of SMI (more than 50% of residents with a diagnosis of schizophrenia or bipolar) and compared them to those below this cutoff. High volume NHs grew from 1.00% (161) of all NHs in 2000 to 3.10% (461) in 2021. While most states gained one or fewer high volume NHs, California gained 54, Illinois 44, Missouri 42, and Ohio 35, suggesting state policies play a role. High volume facilities were fundamentally different from other facilities. Wilcoxon rank sum tests and Pearson’s Chi-squared test of the 2021 data showed significantly (p< 0.001) lower acuity and higher restraint use, 13 year lower average resident age, 0.21, 0.13, and 0.29 fewer registered nurse, licensed practical nurse, and certified nursing assistant hours per resident day respectively, 20% fewer female residents, 16% fewer White residents, 7% fewer residents with dementia, 22% more residents on Medicaid and 22% more facilities being for-profit. More investigation is needed to determine if high volume NHs offer appropriate care for people with SMI, or if they are warehouses for inappropriate segregation from community living.

